# Collateral Rescue, Arterial Burden: Untreated Takayasu Arteritis and its Long-Term Complications

**DOI:** 10.31138/mjr.230524.cra

**Published:** 2025-01-22

**Authors:** Ikwinder Preet Kaur, Steven Morales-Rivera, Victoria Cuello, Gurjit S. Kaeley

**Affiliations:** 1 Department of Rheumatology;; 2 Department of Radiology; University of Florida College of Medicine, Jacksonville, FL, United States of America; 3 Department of Internal Medicine, University of Texas, Rio Grande Valley School of Medicine, Edinburg, TX, United States of America

**Keywords:** Takayasu arteritis, aorta, inflammation, hypertension

Takayasu Arteritis (TA) is a large vessel granulomatous vasculitis that primarily targets the aorta and its major branches. The chronic inflammation leads to luminal remodelling, resulting in severe arterial stenosis, complete occlusion, or less commonly, aneurysmal dilatation. The natural history of TA progresses through three phases: a systemic inflammatory phase, a vascular phase, and a fibrotic phase.^1^

This case report explores the long-term sequelae of untreated TA and highlights the importance of early diagnosis and treatment.

A 51-year-old Albanian female with a past medical history of untreated TA presented for evaluation of high blood pressure. The patient reported being asymptomatic, denying any constitutional symptoms, current vision issues, limb claudication, syncope, dizziness, mental status changes, or focal neurological deficits. She works as a housekeeper and has not noticed any change in her functional capacity. She reported a previous diagnosis with TA in 2007 based on clinical finding of asymmetric blood pressure and imaging studies (records unavailable); however, she never received treatment with steroids or other immunosuppressants. Physical examination revealed asymmetric blood pressure readings in all four extremities, with significantly higher readings in the lower limbs compared to the upper limbs (Right lower extremity 198/94 mm Hg, left lower extremity 204/87 mm Hg, right upper extremity 97/58 mm Hg, left upper extremity 85/54 mm Hg). A left carotid bruit and diminished upper extremity pulses were also noted. Neurological and ophthalmic examinations were unremarkable. Laboratory tests were within normal limits. Upper extremity Doppler ultrasound was normal bilaterally. CTA of the neck, chest, and abdomen demonstrated severe stenosis of the great vessels and subclavian arteries bilaterally, along with occlusion and severe stenosis of the aortic arch. MRA of the neck, chest, and abdomen confirmed these findings. No active inflammation (post-contrast enhancement) was observed (**Figures 1–5**). No significant abnormalities were noted in the abdominal aorta or its branches. Cardiac ECHO was unremarkable. Given the absence of active vasculitis on MRA, the patient was monitored without initiating immunosuppression. Vascular surgery recommended non-operative management. The patient also strongly favoured a conservative approach.

**Figure 1.**
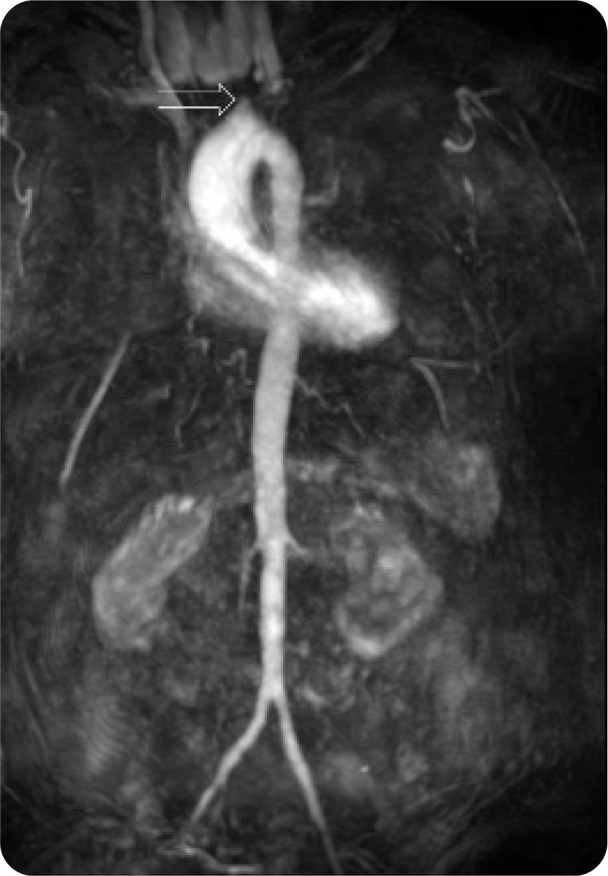
MRA 3D MIP reformat of the aorta showing lack of opacification of great vessels. White arrow showing the origin of the right brachiocephalic artery with lack of opacification distally.

**Figure 2.**
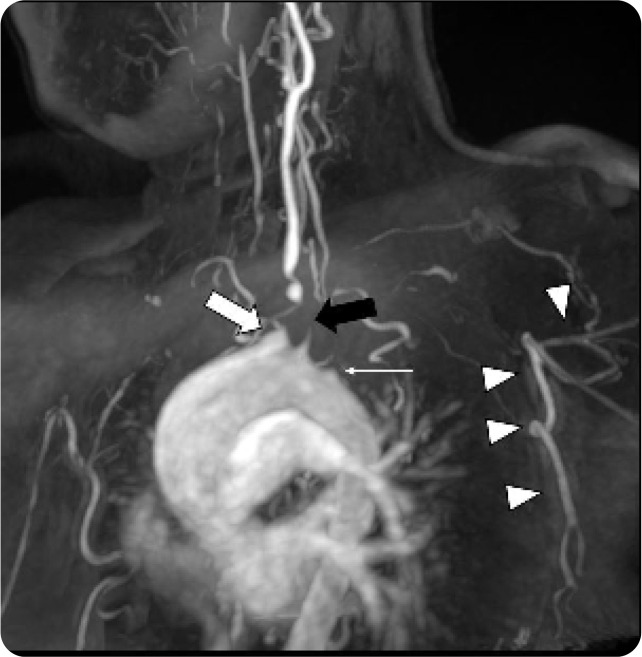
MRA Neck 3D reformat showing occlusion of the right brachiocephalic (thick white arrow) and left subclavian (thin white arrow) arteries as well as severe stenosis of the left common carotid artery (thick black arrow) with good distal opacification. Additionally, collateral circulation through the posterior chest wall supplying the left upper arm is partially seen (white arrow heads).

**Figure 3.**
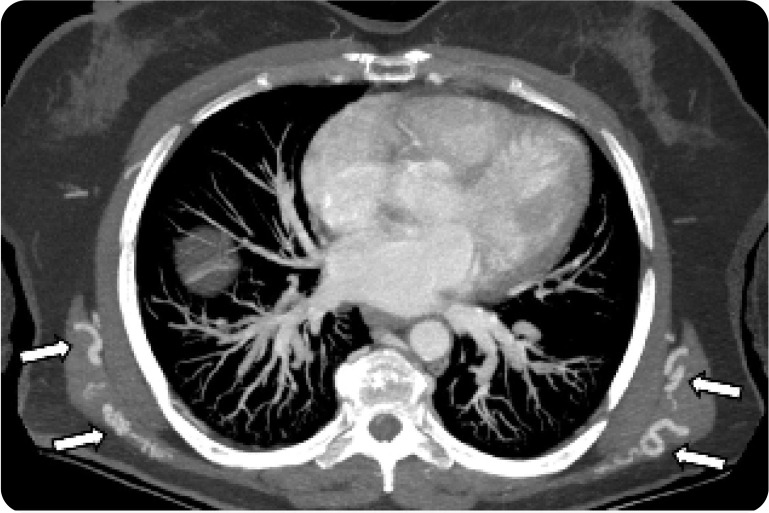
Axial CT Chest MIP reformat showing large collateral circulation arising from intercostal arteries coursing through the posterior chest wall (white arrows). These collaterals will go and reconstitute/supply the upper extremities circulation (not shown).

**Figure 4.**
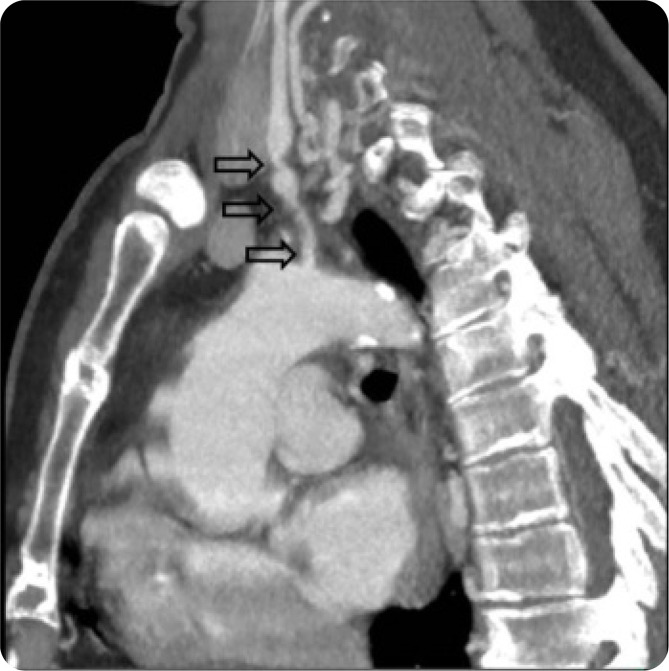
Sagittal CTA chest reformat focusing on the left common carotid artery showing multifocal stenosis (black arrows).

**Figure 5.**
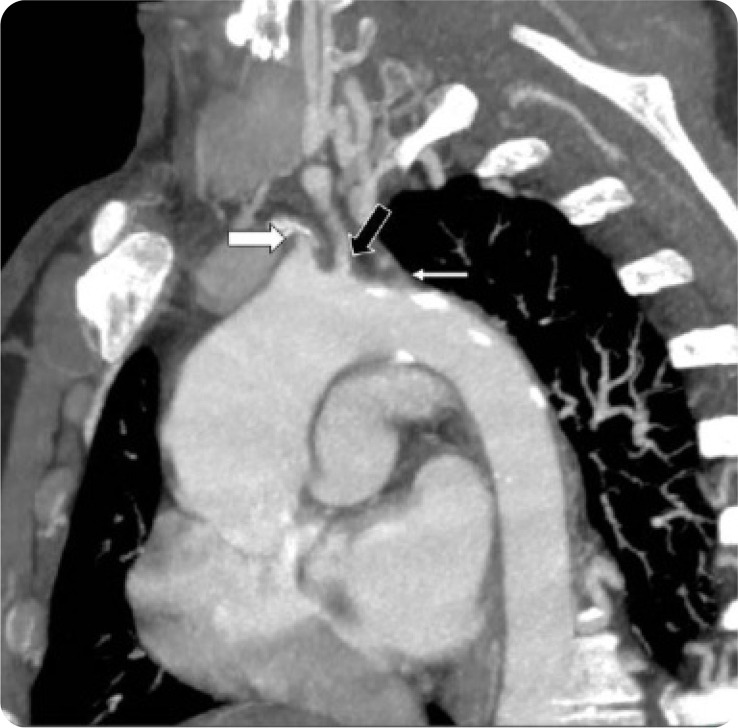
CT Chest MIP “candy cane” reformat demonstrating occlusion of the right brachiocephalic (thick white arrow) and left subclavian (thin white arrow) arteries and severe stenosis of the left common carotid artery (thick black arrow).

The lack of active inflammation on MRA suggests the disease may have transitioned into a burnt-out or fibrotic phase. However, the presence of severe stenosis and occlusions signifies significant long-term vascular damage, increasing the risk of complications such as myocardial infarction, heart failure, aortic regurgitation, stroke, and limb ischemia. Subramanyan et al. reported that severe hypertension, significant functional disability, and evidence of cardiac involvement were strong predictors of adverse events during follow-up in TA patients.2 Long-term data on outcomes of asymptomatic patients is limited. However, TA is known to be a relapsing and remitting disease, with a large French study reporting relapse rates as high as 42% in the first five years.3 Therefore, although the patient is asymptomatic, likely due to significant collateralisation developed over the years, close monitoring and repeat imaging is crucial. Progression of stenosis in the carotid or vertebral arteries can be catastrophic. Surgical intervention in TA is typically reserved for select cases due to the increased risk of complications and technical challenges.4 The patient follows with cardiology for hypertension management, with a permissive hypertension strategy for maintaining cerebral perfusion. She was offered total aortic arch replacement, which she declined at this time. This case exemplifies the potential long-term sequelae of untreated Takayasu Arteritis. Early diagnosis and appropriate treatment are essential to prevent these potentially life-threatening sequelae.

## KEY CLINICAL MESSAGE

Untreated Takayasu Arteritis can silently progress to severe arterial stenosis, even in absence of active inflammation. Early diagnosis and intervention are essential in Takayasu Arteritis to prevent severe, potentially life- or organ-threatening complications.

## CONSENT

Written informed consent was obtained from the patient to publish this report in accordance with the journal’s patient consent policy.

## ETHICS STATEMENT

I confirm that the manuscript has been submitted solely to this journal and is not published, in press, or currently submitted elsewhere.

## CONFLICTS OF INTEREST

The authors declare no conflicts of interest.

## FUNDING

No funding available.
